# Imaging features of lung cancer with cystic airspaces: clinical utility, challenges, and perspectives

**DOI:** 10.3389/fonc.2026.1764251

**Published:** 2026-05-26

**Authors:** Mengyue Hu, Jinbao Feng, Xiaonan Shao, Yunmei Shi, Jianxiong Gao, Yaoting Zhu, Yuhao Fan, Yan Sun, Zhenxing Jiang, Rong Niu, Qianyun Wang

**Affiliations:** 1 Department of Nuclear Medicine, Changzhou Key Laboratory of Molecular Imaging, The Third Affiliated Hospital of Soochow University, The First People′s Hospital of Changzhou, Institute of Clinical Translation of Nuclear Medicine and Molecular Imaging, Soochow University, Changzhou, China; 2 Department of Radiology, The Third Affiliated Hospital of Soochow University, The First People′s Hospital of Changzhou, Changzhou, China; 3 Department of Thoracic Surgery, The Third Affiliated Hospital of Soochow University, The First People′s Hospital of Changzhou, Changzhou, China

**Keywords:** computed tomography, cystic airspaces, lung cancer associated with cystic airspaces, lung neoplasm, positron emission tomography - computed tomography

## Abstract

Lung cancer is a leading cause of cancer-related deaths worldwide and poses a significant health burden. Early diagnosis is critical for reducing lung cancer mortality. Lung Cancer with Cystic Airspaces (LCCA) is a distinct morphological subtype of lung cancer, characterized on imaging by cystic regions accompanied by solid components and/or ground-glass opacities. LCCA is relatively rare, with an incompletely understood pathogenesis, marked heterogeneity, and complex imaging manifestations. It accounts for approximately one-quarter of lung cancer cases that are missed or diagnosed late. With the widespread implementation of lung cancer screening, the detection rate of LCCA is increasing and has drawn growing attention. However, current research is limited by small sample sizes and predominantly retrospective study designs, posing significant clinical challenges in LCCA management. This article reviews the definition and underlying mechanisms of LCCA, systematically summarizes the clinical value of noninvasive imaging in diagnosis, classification, invasiveness prediction, staging, and prognosis assessment of LCCA, and discusses the limitations of existing studies as well as future research directions and challenges. The goal is to provide a theoretical basis for optimizing early lung cancer screening pathways and developing individualized intervention strategies, which holds important clinical significance for reducing lung cancer-related mortality.

## 1 Introduction

Lung cancer is the most common malignancy worldwide and the leading cause of cancer-related deaths (1). While early-stage lung cancer is associated with a favorable prognosis, advanced stages have a poor outlook, with a 5-year survival rate of only 5% (2). In recent years, increasing attention has been given to a distinct morphological subtype of lung cancer known as lung cancer associated with cystic airspaces (LCCA) (3). Studies have shown that LCCA differs from conventional solid or ground-glass nodule-type lung cancers in terms of its pathological mechanisms, imaging features, and prognosis (4). Epidemiological data indicate that LCCA accounts for approximately 1–4% of lung cancer cases (5–7), with a higher incidence in males (8, 9). The predominant histological type is adenocarcinoma, comprising about 80–88% of cases (4, 7, 10). LCCA is characterized by significant heterogeneity and a relatively poor 5-year survival rate (4, 11–13). These features suggest that LCCA may possess a unique tumor microenvironment and evolutionary pattern, warranting further in-depth investigation.

With the widespread use of low-dose computed tomography (LDCT) in lung cancer screening, the detection rate of LCCA has significantly increased (14). However, its clinical diagnosis remains highly challenging. First, the imaging features of LCCA often overlap with those of benign conditions such as pulmonary bullae, lung cysts, and pulmonary tuberculosis (15), making it challenging for radiologists to distinguish LCCA from these benign lesions. In the NELSON lung cancer screening trial, LCCA accounted for approximately 22.7% of false-negative cancers, highlighting the difficulty of early recognition of this rare morphological subtype (16, 17). Therefore, improving radiologists’ ability to identify small-sized LCCA on high-resolution computed tomography (HRCT) before lesion progression is of paramount importance. Second, current diagnostic and treatment guidelines lacks standardized protocols for managing LCCA. Due to its rarity, LCCA has not yet been incorporated into most lung nodule management guidelines. To date, only the Lung CT Screening Reporting and Data System (Lung-RADS) 2022 classification developed by the American College of Radiology (ACR) has preliminarily incorporated cystic nodules into its management recommendations (18). Furthermore, the thin-walled cavities characteristic of LCCA pose limitations for invasive procedures such as percutaneous lung biopsy, which are associated with a high false-negative rate and increased risk of pneumothorax (12, 19). Therefore, noninvasive imaging remains the primary method for preoperative evaluation of LCCA (12).

This article provides a systematic review of recent advances in imaging studies of LCCA, with an in-depth discussion of the current status and progress of various imaging modalities in the qualitative diagnosis, pathological classification, staging, prognosis, and gene expression profiling of LCCA. Distinct from previous reviews, this article offers three key novelties: a comprehensive synthesis of CT-based classification systems with direct correlations to clinical outcomes such as lymph node metastasis, genetic mutations, and prognosis; a critical appraisal of the controversial role of PET/CT in LCCA, highlighting its subtype-dependent metabolic heterogeneity; and a forward-looking perspective on emerging techniques (radiomics, habitat imaging, novel tracers) specifically for lesion characterization after HRCT detection. The aim is to offer more comprehensive diagnostic and prognostic references for clinical practice, thereby improving the early diagnosis and treatment of LCCA.

## 2 Literature search strategy

**Figure 1:**
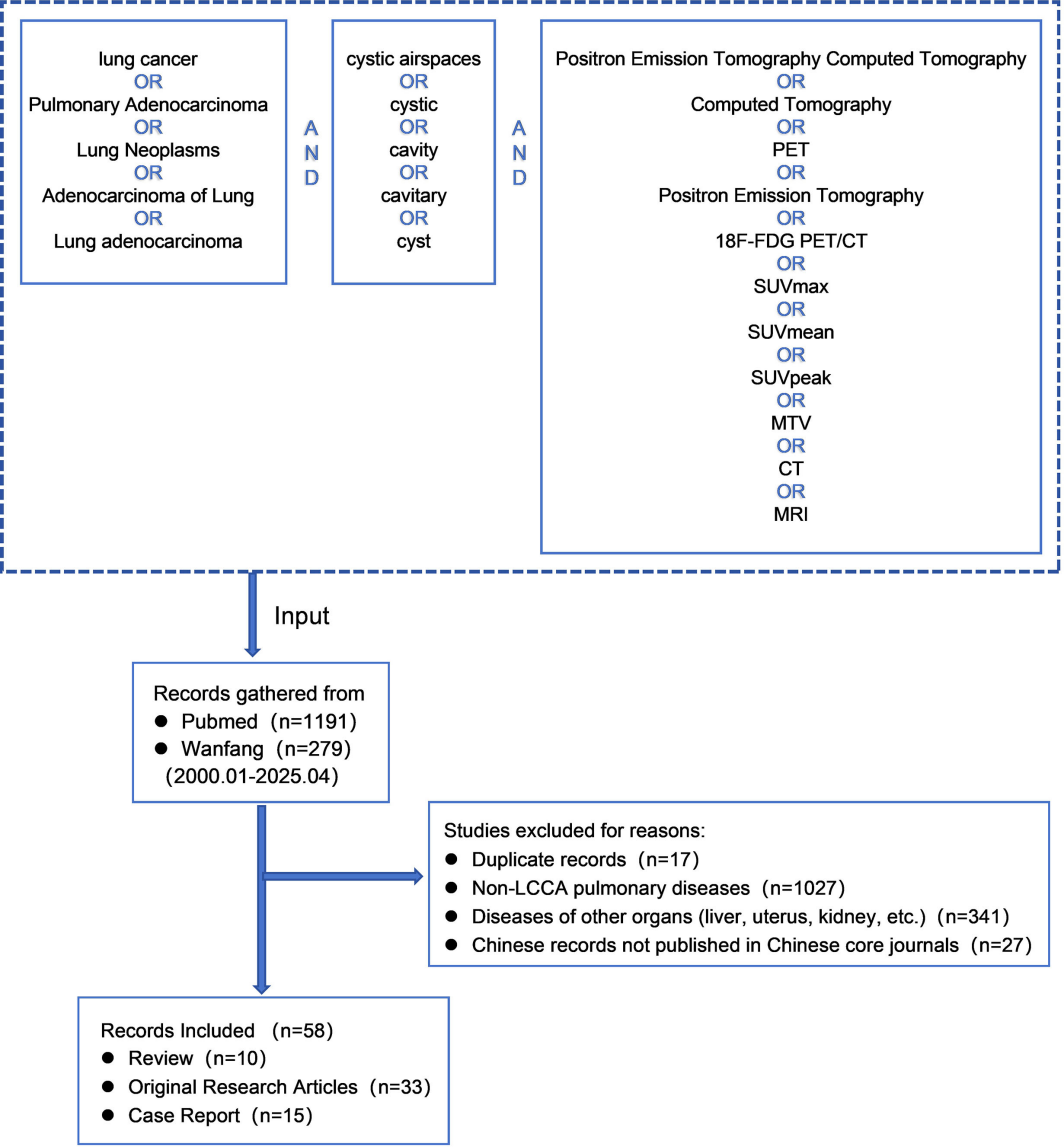
Flow chart of the identification of included studies.

This review systematically examined literature related to LCCA published between January 2000 and April 2025 in the PubMed and Wanfang databases (**Figure 1**). The search strategy was as follows: ((“Adenocarcinoma of Lung”[Mesh]) OR (“Lung Neoplasms”[Mesh]) OR (Lung adenocarcinoma[Title/Abstract]) OR (Lung cancer[Title/Abstract]) OR (Pulmonary Adenocarcinoma[Title/Abstract])) AND ((cystic airspaces[Title/Abstract]) OR (cystic[Title/Abstract]) OR (cavity[Title/Abstract]) OR (cavitary[Title/Abstract]) OR (cyst[Title/Abstract])) AND ((“Positron Emission Tomography Computed Tomography”[Mesh]) OR (Computed Tomography[Title/Abstract]) OR (PET[Title/Abstract]) OR (Positron Emission Tomography[Title/Abstract]) OR (^18^F-FDG PET/CT[Title/Abstract]) OR (SUVmax[Title/Abstract]) OR (SUVmean[Title/Abstract]) OR (SUVpeak[Title/Abstract]) OR (MTV[Title/Abstract]) OR (CT[Title/Abstract]) OR (MRI[Title/Abstract])). A total of 1,470 articles were retrieved. After excluding 1412 articles (duplicates: n=17; non-LCCA pulmonary diseases: n=1,027; diseases of other organs: n=341; Chinese publications outside core journals: n=27), 58 relevant publications were included in the final review. Among these, there were 33 original research articles (all retrospective studies), 10 review articles, and 15 case reports. The Newcastle-Ottawa Scale was employed to evaluate the methodological quality of the 33 included retrospective studies. The majority of these studies were of moderate and high quality, with retrospective design and small sample sizes being the primary limitations.

## 3 Definition and mechanisms of LCCA

LCCA was first reported by Anderson et al. in 1954 (20). Over time, it has been referred to by various names, such as thin-walled cavitary lung cancer, carcinoma of the bronchus presenting as thin-walled cysts, and lung carcinoma associated with bullous lung disease. Among these, “LCCA” has become the most widely used term in recent years (12, 20, 21). The internationally recognized definition of LCCA is: malignant tumors with cavities or cysts formed in the lung parenchyma (22). On thin-section CT, it typically presents as air-containing spaces accompanied by mural nodules or ground-glass opacities. However, the exact carcinogenic mechanism behind cystic airspaces (CAs) formation remains unclear. Currently, several hypotheses have been proposed based on imaging–pathology correlations, focusing mainly on airway obstruction, tissue necrosis, and structural remodeling of the lung. These include: ① Airway or microvascular obstruction: The “check-valve” mechanism caused by small peripheral tumors obstructing distal airways, or the “ball-valve phenomenon” due to external compression by fibrotic tissue produced by tumor cells. Alternatively, ischemic dilatation may occur due to microvascular obstruction of the bronchioles, leading to gas retention within the tumor and eventual CAs formation (10, 23–25). ② Liquefaction and necrosis: Solid lesions may undergo necrosis and liquefaction, with subsequent bronchial drainage allowing air to enter and form CAs (26). ③ Secondary to structurally weakened alveoli: In patients with a history of chronic smoking, destruction of alveoli and terminal bronchioles compromises ventilation and clearance, promoting the accumulation of carcinogens and contributing to CAs development (6, 7, 12, 25, 27). Genetic, inflammatory, and other related factors (28, 29). Most studies support the “check-valve” mechanism involving peripheral small airways. However, this mechanism does not adequately explain LCCA cases arising in central lung zones, suggesting the possibility of a central airway origin or other unrecognized mechanisms (30). Nonetheless, these proposed mechanisms are largely based on retrospective data, and direct evidence from prospective studies at the molecular and pathological levels is still lacking.

## 4 CT features and applications of LCCA

### 4.1 CT features of LCCA

CT is the preferred and most important imaging modality for the diagnosis and differential diagnosis of LCCA. Studies have found that 67.5% of LCCA cases are located in the peripheral zones of the lungs, with no clear predilection for specific lobes (7, 25, 31, 32). Research by Shen et al. (31) and Xue et al. (23) indicates that typical LCCA lesions often exhibit common malignant signs such as irregular margins, spiculation, vascular convergence, pleural indentation, and lobulation. In addition, LCCA possesses distinctive imaging features, including mural nodules and internal septations. Internal septations are thought to be fibrous structures formed by tumor cells, bronchi, or blood vessels, and have been reported in more than half of LCCA cases (10, 33). Mendoza et al. (4), through a meta-analysis of 8 studies involving a total of 341 patients, systematically summarized the CT imaging characteristics of LCCA. The most common feature was non-uniform cyst walls (91.2%), followed by the presence of a nodular component (64.0%), unilocular cysts (63.6%), thick walls (37.4%), and irregular margins (37.3%). To facilitate accurate assessment of LCCA, some studies have proposed that, similar to ground-glass nodules, thin-section CT (slice thickness ≤ 1.5 mm) and HRCT should be used for both diagnosis and follow-up (34, 35). Additionally, multiplanar reconstruction is recommended to better visualize the relationship between the cystic and non-cystic components of the tumor (21).

Early malignant signs of LCCA are often atypical, and the disease is frequently misdiagnosed or missed due to its resemblance to benign conditions such as pulmonary bullae, lung cysts, or infections. At present, research on the malignant risk assessment of cystic lung lesions is limited. Yao et al. (36) conducted a retrospective study involving 129 patients with cystic pulmonary nodules (92 malignant and 37 benign cases). Multivariate regression analysis identified mural nodules, spiculation, irregular cyst wall morphology, and multilocular cysts as independent predictors of LCCA. The nomogram model achieved an area under the curve (AUC) of 0.874, indicating good predictive performance. Regular CT follow-up is essential for further differentiation of cystic pulmonary nodules. Malignant LCCA typically shows cyst enlargement or contraction accompanied by mural nodule formation, cyst wall thickening, or complete solidification, whereas benign lesions tend to remain stable or exhibit minimal changes (10, 37).

**Figure 2:**
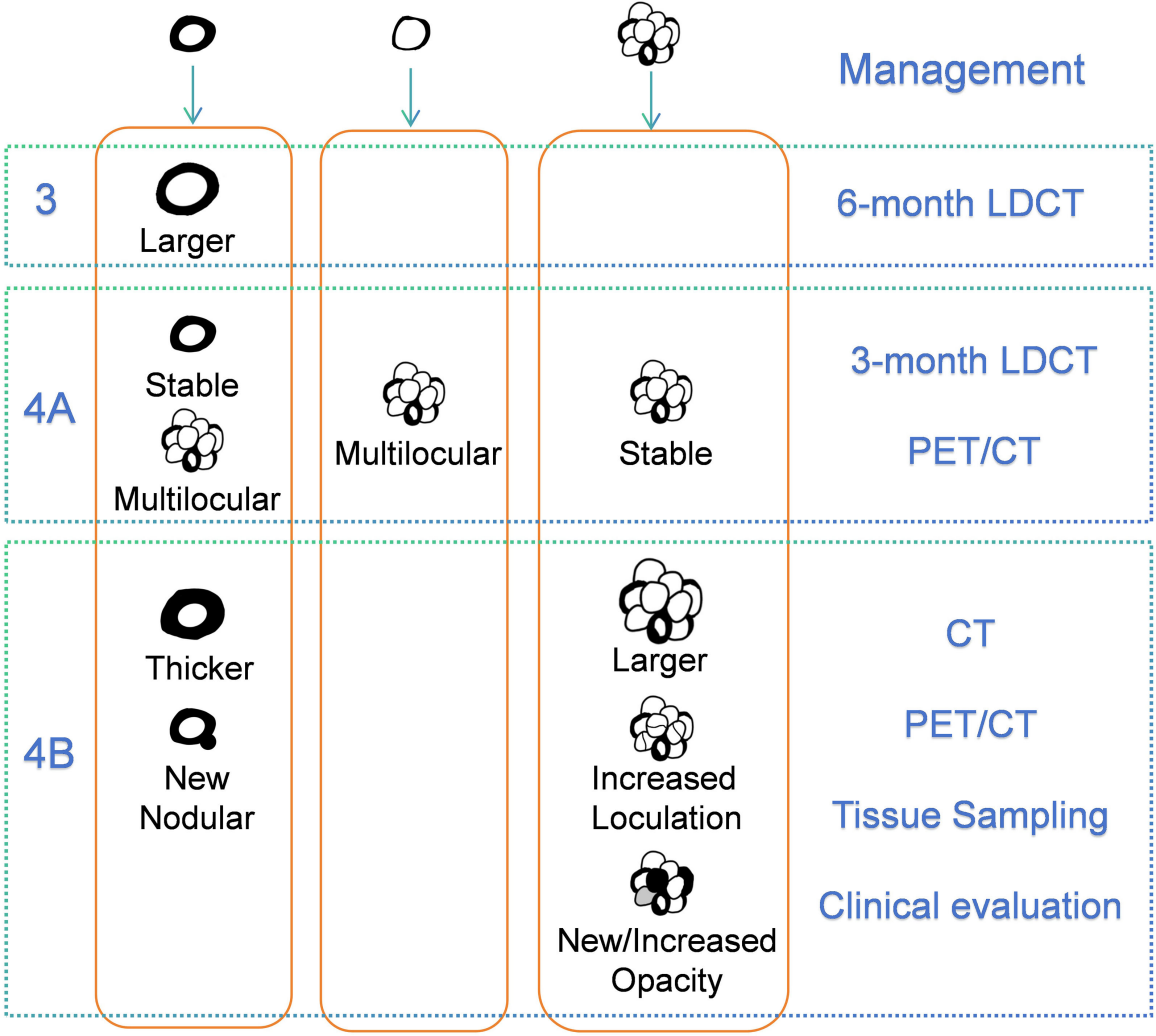
Atypical pulmonary cysts in lung RADS v2022.

Lung-RADS is a standardized radiological classification system for pulmonary lesions, categorizing them into five levels (0 to 4) based on their malignancy risk. In the 2022 update, Lung-RADS introduced the definition of an *atypical lung cyst* (**Table 1** (18) and **Figure 2**), referring to a cyst with a wall thickness of 2 mm or more, featuring uniform or asymmetrical morphology, which may be unilocular or multilocular, and may contain mural nodules. Thick-walled cysts with an increase in the average diameter of the cystic component are classified as Lung-RADS 3, with a recommendation for follow-up using LDCT after 6 months. Thick-walled cysts and multilocular cysts are classified as Lung-RADS 4A. It is recommended to perform LDCT after 3 months. If the solid component is ≥ 8 mm, PET/CT follow-up may be considered. If a thick-walled cyst shows wall thickening or an increased number of mural nodules, or if a multilocular cyst increases in size, exhibits more internal septations, or is accompanied by newly appearing or enlarging adjacent opacities (such as nodules, ground-glass opacities, or consolidations), it is upgraded to Lung-RADS 4B, with diagnostic evaluation (such as biopsy) recommended. Thin-walled cysts and multiple pulmonary cysts without malignant features are not specifically classified in the guideline. It is worth noting that cysts with nodular wall thickening (e.g., thick-walled cysts) can be difficult to distinguish from thin-walled cysts with mural nodules on imaging. The guidelines recommend assigning the higher category (e.g., category 4B) in such cases. Since the malignancy risk of atypical lung cysts remains uncertain, risk stratification beyond category 4B is not routinely applied in Lung-RADS recommendations. Therefore, clinical decision-making should rely on dynamic imaging changes and multidisciplinary discussion.

**Table 1:** Atypical pulmonary cysts in lung RADS v2022.

Lung-RADS	Description	Management
3	Growing cystic component (mean diameter) of a thick-walled cyst	6-month LDCT
4A	Thick-walled cyst OR multilocular cyst at baseline OR thin-or thick-walled cyst that becomes multilocular	3-month LDCT; PET/CT may be considered if there is a ≥ 8 mm (≥ 268 mm^3^) solid nodule or solid component
4B	Thick-walled cyst with growing wall thickness/nodularity OR growing multilocular cyst (mean diameter) OR multilocular cyst with increased loculation or new or increased opacity (nodular, ground glass, or consolidation)	Diagnostic chest CT with or without contrast; PET/CT may be considered if there is a ≥8mm (≥ 268 mm^3^) solid nodule or solid component; tissue sampling; and/or referral for further clinical evaluation

LDCT, low-dose CT; Lung-RADS, Lung CT Screening Reporting and Data System.

### 4.2 Classification systems of LCCA

**Figure 3:**
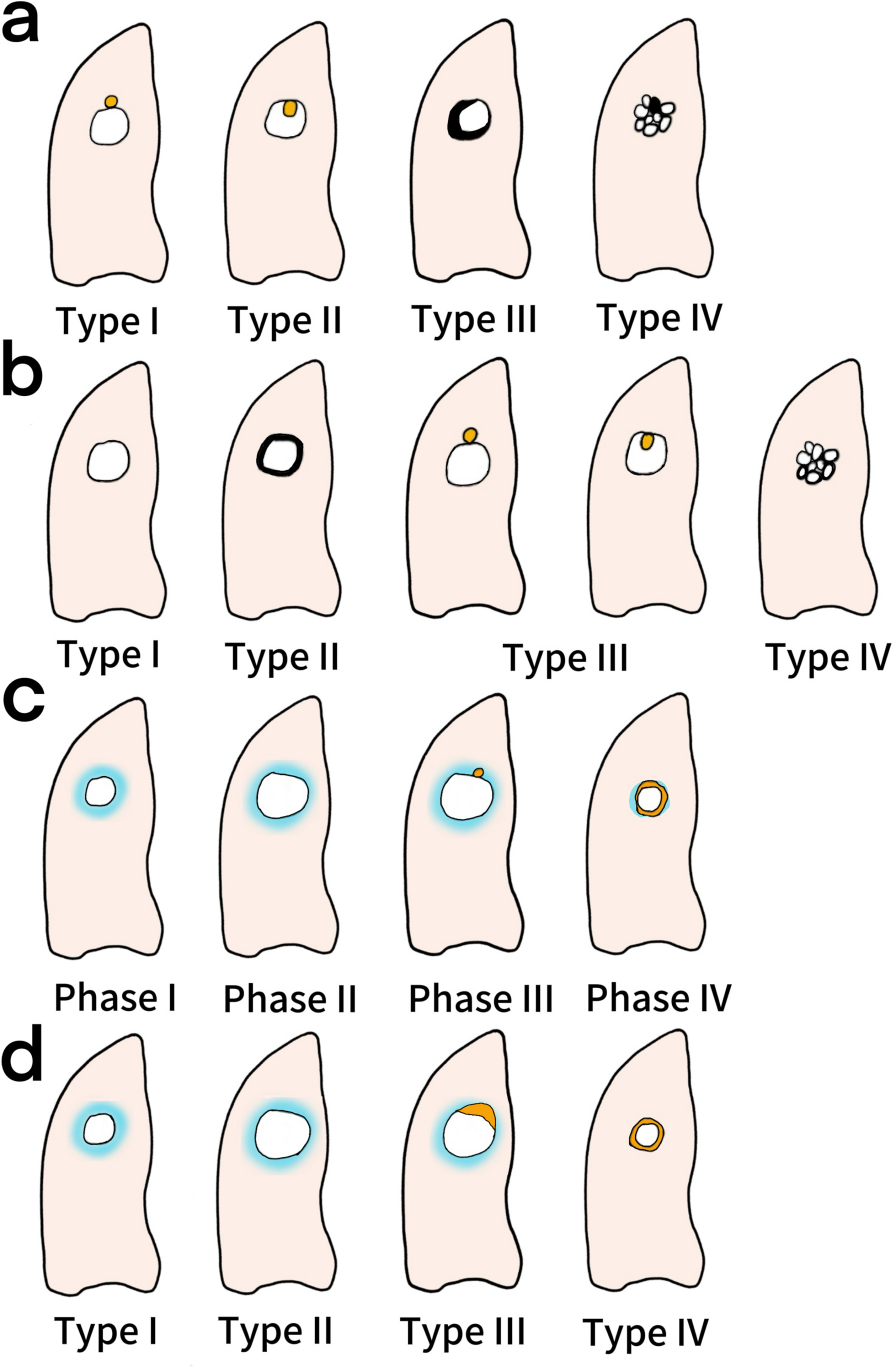
Classification systems of LCCA. **(a)** Mascalchi et al. (32) classification: Type I, exophytic; Type II, endophytic; Type III, soft tissue density extending along the cyst wall; Type IV, soft tissue density intermixed within clusters of CAs. **(b)** Shen et al. (31) classification: Type I, thin-walled, Type II, thick-walled, Type III, mural nodule type, Type IV, mixed type. **(c)** Jung et al. (39) classification: Phase I, CAs appear within non-solid nodule; Phase II, CAs enlarge; Phase III, solid component appears at the CAs border; Phase IV, cyst wall thickens, cyst size decreases, and solid component gradually encloses the cyst. **(d)** Zhu et al. (14) classification: Type I, Pure ground-glass opacity with a cyst size < 6.5 mm; Type II, Pure ground-glass opacity with a cyst size ≥ 6.5 mm; Type III, Cyst with a part-solid nodule; Type IV: Cyst with a solid nodule.

To standardize the diagnostic and therapeutic approach to LCCA and to predict its biological behavior, several classification systems have been developed in recent years based on the CT morphological characteristics and dynamic evolution of LCCA. In 2006, Maki et al. (38) first proposed a classification system for LCCA, which was later described and refined by Mascalchi et al. in 2015 (32) (**Figure 3a**). The classification includes: Type I: Nodule or mass extruding from the wall of the CAs; Type II: Nodule or mass confined within the CAs; Type III: Soft tissue density extending along the wall of the CAs; Type IV: Soft tissue density intermixed within clusters of CAs. Building on this, Shen et al. (31) proposed another classification system based on Mascalchi’s framework (32) (**Figure 3b**): Type I: Thin-walled type, with an average wall thickness < 2 mm; Type II: Thick-walled type, with an average wall thickness between 2 mm and 4 mm; Type III: Mural nodule type, with either endophytic or exophytic nodules; Type IV: Mixed type, where multiple cysts are interspersed within a soft tissue cluster. However, no studies have yet established a definitive association between morphological subtypes and the prognosis of LCCA. The different morphologies described in these systems may represent different phases of a continuous pathological process. Based on sequential CT scans, Jung et al. (39) proposed a stepwise progression model of subsolid LCCA to capture these morphological changes over time (**Figure 3c**): Phase I: CAs appear in the middle of non-solid nodules; Phase II: The CAs grow; Phase III: A solid component appears on the border of the CAs; Phase IV: The cyst wall begins to thicken, the cyst size decreases, and solid components gradually enclose the cyst. Building on Jung’s classification (39), Zhu et al. (14) proposed a modified classification system that offers greater clinical value in predicting pathological invasiveness (**Figure 3d**): Type I: Pure ground-glass opacity with a cyst size < 6.5 mm; Type II: Pure ground-glass opacity with a cyst size ≥ 6.5 mm; Type III: Cyst with a part-solid nodule; Type IV: Cyst with a solid nodule.

At present, most studies adopt the classification proposed by Shen et al., which is based on cyst wall thickness and nodule characteristics. This system is widely used due to its intuitive nature and practical applicability. For Shen type I and II lesions with a wall thickness ≤ 4 mm, CT surveillance may be appropriate, whereas type III and IV lesions require clinical consultation regardless of nodule size. In contrast, Jung’s classification, which evaluates morphological changes through serial CT scans, allows for a more comprehensive assessment of the tumor’s biological behavior. The operability of Shen’s classification, the dynamic sensitivity of Jung’s classification, and the risk-predictive capability of Zhu’s classification may together serve as important foundations for future clinical management of LCCA.

### 4.3 Invasiveness assessment of LCCA

Histological studies have shown that LCCA is predominantly adenocarcinoma (80%–88%), referred to as lung adenocarcinoma associated with cystic airspaces (LACA). At the time of diagnosis, more than 90% of LACA cases are invasive adenocarcinomas (4, 7, 10, 31), with a 5-year survival rate significantly lower than that of minimally invasive adenocarcinoma or pre-invasive lesions (40). Therefore, accurate preoperative identification of LACA invasiveness is crucial for selecting appropriate treatment strategies and predicting patient prognosis.

Wang et al. (41) conducted a retrospective analysis of 541 patients with pathologically confirmed lung adenocarcinoma, including 87 cases of LACA and 454 cases of non-LACA. The results showed that the presence of CAs itself was an independent predictor of invasive adenocarcinoma (odds ratio [OR]: 3.220). Moreover, the incidence of invasive adenocarcinoma was higher in LACA with multiple CAs compared to those with a single cystic space (72.2% vs. 47.1%). Zhang et al. (34) analyzed HRCT data from 176 cases of LACA with diameters ≤ 3 cm. Their findings demonstrated that the density of the wall component was positively correlated with tumor invasiveness. Additional important imaging predictors of invasive LACA included the presence of septa within the cyst, CAs diameter, wall thickness, and the proportion of thin-walled structures. Zhu et al. (14) retrospectively analyzed 265 lesions from 252 LACA patients, including 182 invasive lesions and 83 pre-invasive or minimally invasive lesions. Independent risk factors included multiple CAs (OR = 5.599), irregular shape of CAs (OR = 3.236), larger tumor size [(23.9 mm ± 10.3 mm) vs. (11.8 mm ± 4.6 mm), OR = 1.281], and higher CT attenuation values [(−127.1 HU ± 246.7 HU) vs. (−557.3 HU ± 192.1 HU), OR = 1.007]. The predictive model achieved an AUC of 0.964, indicating excellent predictive performance. In addition, LCCA classification systems also provide value in predicting invasiveness. The study by Zhu et al. (14) demonstrated that types III and IV in both the Shen and Zhu classification systems were significantly associated with invasive LACA. In summary, these studies suggest that for cystic nodules, the presence of multiple cystic spaces, irregular shape, and higher CT attenuation (≥ –127 HU) should alert clinicians to a high suspicion of invasive adenocarcinoma, warranting early intervention rather than observation.

### 4.4 Prediction of LCCA growth patterns

As the predominant histological subtype of LCCA, LACA exhibits several growth patterns, including lepidic, acinar, papillary, micropapillary, and solid types. Based on the predominant growth pattern and its proportion, LACA can be classified into three grades, corresponding to well, moderately and poorly degrees of differentiation. Different imaging classifications have been found to correlate with specific LACA growth patterns or differentiation types. Jung et al. (39) reported that phase II LACA was predominantly of the lepidic type (59.5%), while phases III and IV were mainly acinar (37.5% and 30.2%, respectively). As LACA progressed from phase II to phase IV, the proportion of lepidic growth decreased (from 59.5% to 11.8%), whereas the proportions of solid and micropapillary components increased (from 0% to 15.0% and from 1.3% to 18.6%, respectively). In Shen et al.’s (31) classification system, LACA in types I and II exhibited equal proportions of well-differentiated and moderately/poorly (M/P) differentiated tumors (both 50%). In contrast, types III and IV were predominantly composed of M/P differentiated LACA, with type III showing the highest proportion of M/P differentiation (85.0%). Multivariate analysis identified type III, partial or complete solid components in the cyst wall, and an irregular inner surface of the cyst as independent predictors of M/P differentiated LACA. These findings are consistent with those reported by Ma et al. (9).

### 4.5 Lymph node and distant metastasis

Lymph node involvement and distant metastasis are critical for tumor staging and determining treatment strategies. However, to our knowledge, there are currently no systematic studies using CT to preoperatively predict or assess lymph node or distant metastasis in LCCA. Preliminary insights can only be drawn from a few small-scale descriptive studies, which may serve as a reference for future research into N and M staging of LCCA. Xue et al. (23) reported that among 18 patients with thin-walled LCCA (wall thickness between 1 mm and 4 mm, presumably corresponding to Shen type I–II), 16 had neither lymph node nor distant metastases, while 2 cases developed pulmonary metastases. In the study by Farooqi et al. (6), among 26 patients with Shen type III LCCA, 5 (19.2%) had lymph node metastases, with 2 cases of N1 disease and 3 cases of N2. No distant metastases were observed. Ma et al. (9) conducted a study involving 352 cases of LCCA, in which the overall lymph node metastasis rate was 28.4%. No lymph node involvement was observed in Shen type I. In type II, the rates of N1 and N2 metastasis were both 14.7%. For type III, the N1 and N2 metastasis rates were 13.8% and 15.8%, respectively. In type IV, the rates were 13.3% for N1 and 17.3% for N2. Jung et al. (39) also found that N stage increased with LACA progression. In phase II, no lymph node metastasis was observed. In phase III, the N1 metastasis rate was 3% and N2 was 6.1%, whereas in phase IV, the rates rose to 11.8% for N1 and 29.4% for N2. The patterns of lymph node involvement and distant metastasis in patients with LCCA are summarized in **Table 2**.

**Table 2:** Lymph node and distant metastasis in LCCA.

Author	Year	No. of patients	Classification	N1	N2	Distant metastasis
Jung et al. (39)	2020	60	Jung et al.	Phase II: 0% (0/10) Phase III: 3% (1/33) Phase IV: 11.8% (2/17)	Phase II: 0% (0/10) Phase III: 6.1% (2/33) Phase IV: 29.4% (5/17)	–
Xue et al. (23)	2012	18	–	0% (0/18)	0% (0/18)	11.1% (2/18)
Ma et al. (9)	2022	352	Shen et al.	Type I: 0% (0/16) Type II: 14.7% (16/109) Type III: 13.8% (21/152) Type IV: 13.3% (10/75)	Type I: 0% (0/16) Type II: 14.7% (16/109) Type III: 15.8% (24/152) Type IV: 17.3% (13/75)	–
Shen et al. (31)	2019	123	Shen et al.	0.8% (1/123)	7.3% (9/123)	–
Farooqi et al. (6)	2012	26	Farooqi et al.	7.7% (2/26)	11.5% (3/26)	0% (0/26)
Zhang et al. (34)	2024	176	–	6.5% (10/153) N stage not distinguished		23.9% (16/67)

The above studies suggest a potential correlation between LCCA classification systems and tumor staging. Higher rates of lymph node metastasis have been observed in Shen type III and IV LCCA, as well as in the later phases of the Jung’s classification. This indicates that the likelihood of lymph node involvement and higher tumor stage may increase with the appearance of mural nodules and thickening of the cyst wall. However, current studies are limited by small sample sizes, inconsistent imaging classifications, and variable lesion sizes among enrolled cases. Further research is needed to comprehensively analyze and validate these associations.

### 4.6 Genetic mutation status

The preoperative identification of genetic mutations in LCCA using noninvasive imaging methods can help select patients likely to benefit from targeted therapies and optimize individualized treatment strategies. A meta-analysis by Mendoza et al. (4) indicated that the most common genetic mutations in LCCA were EGFR (37.7%) and KRAS (17.2%), consistent with findings reported by Zheng et al. (13). Ma et al. (9) found heterogeneity in EGFR mutation rates among different Shen classification subtypes, with the highest mutation rate in type III (68.4%), followed by type IV (57.3%), type II (48.6%), and type I (37.5%). Conversely, other studies have reported no significant difference in EGFR mutation rates across LCCA subtypes (31, 42). Jung et al. (39) reported that p53 mutations occurred in up to 36.7% of LACA patients, with a positive correlation between p53 mutation rate and phase: 10% in phase II, 42.4% in phase III, and 41.2% in phase IV. P53 mutation is considered a poor prognostic factor (43). Currently, findings on the genetic mutation landscape of LCCA remain inconsistent. Although different imaging-based classifications may be correlated with specific genetic alterations, further investigation is needed to clarify these associations. The association between the genetic mutation status and imaging classification of LCCA is summarized in **Table 3**.

**Table 3:** Genetic mutation rates of LCCA imaging classification.

Author	Year	No. of patients	EGFR	KRAS	p53	ALK
Mendoza et al. (4)	2021	341 LCCA	37.7% (46/122)	17.2% (21/122)	–	–
Fintelmann et al. (7)	2017	26 LCCA	3.8% (1/26)	53.8% (14/26)	–	–
Jung et al. (39)	2020	60 LACA	–	–	Phase II: 10% (1/10) Phase III: 42.4% (14/33) Phase IV: 41.2% (7/17)	–
Shen et al. (31)	2019	123 LCCA	Type I: 50% (7/14) Type II: 52.4% (11/21) Type III: 57.1% (20/35) Type IV: 46.7% (7/15)	Type I: 0% (0/14) Type II: 10.0% (2/20) Type III: 8.8% (3/34) Type IV: 0% (0/15)	–	Type I: 0% (0/14) Type II: 0% (0/19) Type III: 3.3% (1/30) Type IV: 0% (0/15)
Zheng et al. (13)	2012	165 LCCA	59.8% (64/107)	14.0% (15/107)	–	–
Ma et al. (9)	2022	352 LACA	Shen classification: Type I: 37.5% (6/16) Type II: 48.6% (53/109) Type III: 68.4% (104/152) Type IV: 57.3% (53/75)	–		1.1%

### 4.7 Prognostic evaluation

Currently, studies on the prognostic characteristics of patients with LCCA remain limited. Research has shown that patients with Shen type I LCCA have the longest progression-free survival (PFS), while those with type III have the shortest. The 3-year recurrence-free survival (RFS) rates for Shen types I to IV were reported as 100%, 84%, 77%, and 83%, respectively (5). Ma et al. (9) further validated the prognostic value of the Shen classification. Patients with type I LACA had favorable overall survival (OS), while those with type II had the poorest outcomes. The 5-year OS rates for Shen types I to IV LACA were 100%, 49.4%, 74.6%, and 70.4%, respectively. Multivariate analysis identified age, type II classification, wall thickness, and the presence of a solid wall as independent prognostic factors for OS in LACA patients. Similarly, Watanabe et al. (44) reported that patients with Shen type II LCCA had higher postoperative recurrence and distant metastasis rates compared to those with type I. Jung et al. (39) also demonstrated that the imaging progression phase of LCCA strongly correlates with prognosis: more advanced phases were associated with worse outcomes and increased recurrence risk. Among patients with LACA, the 5-year RFS rates for phases II to IV were 100%, 56%, and 16%, while the corresponding 5-year OS rates were 100%, 70%, and 48%. This study also found that the thicker the solid cyst wall, the higher the recurrence rate. These existing studies provide preliminary evidence linking CT imaging features of LCCA with patient prognosis. However, given the retrospective design and small sample sizes, further validation through large-scale, multicenter prospective cohort studies is warranted. The association between the imaging classification and prognosis of LCCA is summarized in **Table 4**.

**Table 4:** Imaging classification and prognosis in LCCA.

Author	Year	No. of patients	Classification	PFS	OS
Shen et al. (5)	2021	123 LCCA	Shen	3-year RFS rate: Type I: 100% Type II: 84% Type III: 77% Type IV: 83%	–
Jung et al. (39)	2020	60 LACA	Jung	5-year RFS rate: Phase II: 100% Phase III: 70% Phase IV: 48%	–
Ma et al. (9)	2022	352 LACA	Shen	–	5-year OS rate: Type I: 100% Type II: 49.4% Type III: 74.6% Type IV: 70.4%

## 5 Other imaging features and applications

**Figure 4:**
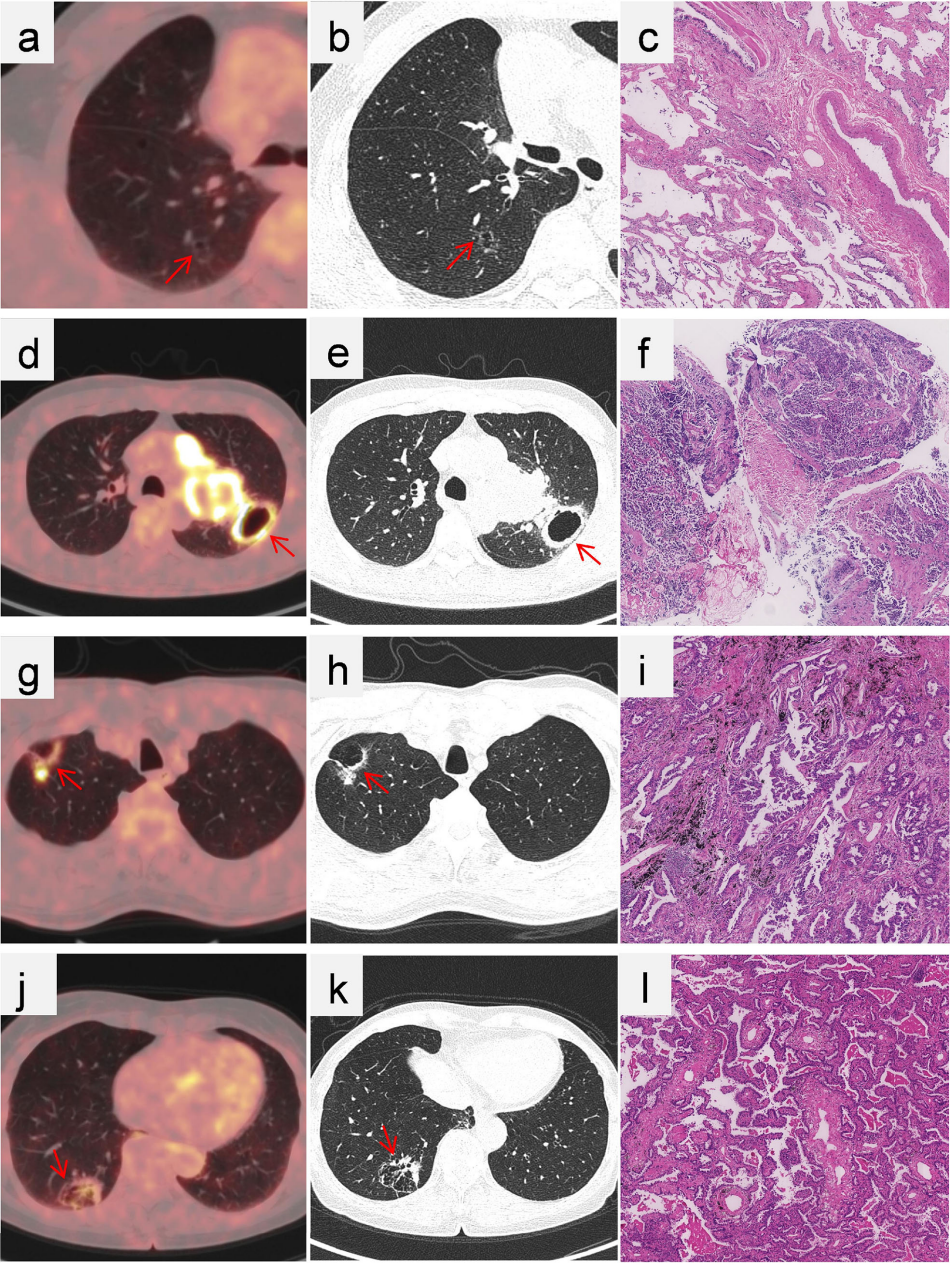
Shen classification of typical cases. A 73-year-old man with Type I LCCA. **(a-c)** An ^18^F-FDG PET/CT image showing no fluorodeoxyglucose uptake (SUVmax = 0.8) (arrow). **(b)** A CT image showing a 1.4 × 0.8 cm cystic ground-glass nodule shadow (arrow). **(c)** Invasive adenocarcinoma (H and E, ×40). A 53-year-old man with Type II LCCA. **(d-f)** An ^18^F-FDG PET/CT image showing strong fluorodeoxyglucose uptake (SUVmax = 17.9) (arrow). **(e)** A CT image showing a 3.8 × 4.4 cm cystic mass shadow (arrow). **(f)** Small cell carcinoma (H and E, ×40). A 64-year-old man with Type III LCCA. **(g-i)** An ^18^F-FDG PET/CT image showing fluorodeoxyglucose uptake (SUVmax=6.6) (arrow). **(h)** A CT image showing a 3.1 × 4.3 cm cystic ground-glass opacity with solid nodular shadows at the edges (arrow). **(i)** Invasive adenocarcinoma (H and E, ×40). A 73-year-old woman with Type IV LCCA. **(j-l)** An ^18^F-FDG PET/CT image showing mild fluorodeoxyglucose uptake (SUVmax=2.6) (arrow). **(k)** A CT image showing a 4.1 × 3.2 cm cystic cavity with soft tissue density shadow at the edge (arrow). l, Invasive adenocarcinoma (H and E, ×40).

PET/CT imaging offers the advantage of integrating functional metabolic data with anatomical structure. The most commonly used radiotracer is fluorine-18 fluorodeoxyglucose (^18^F-FDG), a radiolabeled glucose analog that can be taken up by tumor cells, with uptake increasing in parallel with the Warburg effect (45). ^18^F-FDG PET/CT has been widely used in lung cancer for diagnosis, staging, treatment response evaluation, and prognosis prediction (46–51). Currently, studies on PET/CT imaging in LCCA are scarce and mostly limited to case reports. The reported SUVmax values vary widely, ranging from no uptake to as high as 14.5, reflecting substantial heterogeneity (11, 52–55). Snoeckx et al. (56) suggested that the utility of ^18^F-FDG PET/CT in LCCA may be limited. Several factors may explain this: First, the presence of CAs may lead to reduced FDG uptake (32), especially in LCCA with thin cyst walls or walls composed primarily of ground-glass opacity (57–59). Second, FDG is not a tumor-specific tracer; inflammatory lesions such as cavitary tuberculosis can also exhibit high false-positive uptake, making differential diagnosis challenging (60, 61). Typical cases are shown in **Figure 4**.

Despite the aforementioned controversies, some studies suggest that PET/CT may have value in predicting invasiveness and assessing staging and prognosis of LCCA. Son et al. (57) found that in lung adenocarcinomas predominantly composed of ground-glass opacity, the mean SUVmax of minimally invasive adenocarcinoma (0.86) was significantly lower than that of invasive adenocarcinoma (1.36, P = 0.029). Using a threshold of SUVmax = 1.0, the sensitivity and specificity for differentiation were 61% and 71%, respectively. Given that most LCCA lesions are adenocarcinomas and frequently contain ground-glass components, these findings imply that a higher SUVmax in LCCA may also indicate greater tumor aggressiveness and, consequently, a poorer prognosis. Meanwhile, in terms of staging evaluation, PET/CT is more accurate than CT alone in detecting lymph node and distant metastases. Fintelmann et al. (7) reported that among 30 LCCA cases, 17% had lymph node metastases and 17% were stage IV; Mascalchi et al. (32) found that 50% of patients were diagnosed at an advanced stage (II–IV), with PET/CT playing an important role in detecting distant metastases.

In summary, the diagnostic value of PET/CT imaging in LCCA remains controversial, with few studies addressing its use in evaluating pathological subtypes, invasiveness, staging, or prognosis. However, existing literature suggests that FDG metabolic activity may vary significantly across different LCCA imaging classification and disease stages. For example, a study by Fintelmann et al. (7) indicated that FDG uptake in LCCA may be associated with its classification systems. Most Shen type II and III lesions demonstrated moderate to high FDG uptake. Similarly, in the classification system proposed by Mascalchi et al. (32), type III and IV LCCA showed significant tracer uptake, whereas types I and II exhibited little or no uptake. Further studies are needed to clarify the quantitative associations and clinical relevance of FDG-related metabolic parameters, such as SUVmax, total lesion glycolysis (TLG), and metabolic tumor volume (MTV), with LCCA malignancy and invasiveness.

## 6 Challenges and future perspectives

Despite significant advancements in imaging technologies for the diagnosis and assessment of LCCA, numerous challenges remain to be addressed. First, although various imaging classification systems for LCCA each offer unique advantages, the lack of a unified standard results in marked inconsistencies across studies. This variability limits interobserver agreement in clinical practice, and research linking classification systems to molecular features and prognosis remains confined to small, single-center cohorts, leading to the lack of a precise risk stratification system based on classification. There is an urgent need for multi-center collaboration to develop a more concise and comprehensive classification system that incorporates both static characteristics and dynamic progression, and simultaneously to explore imaging phenotyping based on unsupervised clustering methods to achieve more objective, data−driven automated subtyping. Second, conventional imaging methods have inherent limitations in diagnosing LCCA. For example, a single CT scan often fails to reliably distinguish LCCA from benign lesions. Repeated scans increase unnecessary radiation exposure for patients with benign cystic lesions, and disparities in CT slice thickness across different healthcare facilities further hinder early detection. There is also ongoing debate regarding the measurement of lesion size on CT. Including the cystic portion may overestimate the clinical stage, while focusing only on the solid or ground-glass components may overlook the relationship between tumorigenesis and its radiological features. It is essential to enhance radiologist training in recognizing early-stage LCCA on HRCT in daily clinical practice, which may improve early diagnosis and ultimately save lives. With regard to ^18^F-FDG PET/CT, its sensitivity is often inadequate in LCCA with thin walls or small nodules due to low metabolic activity (57–59). For LCCA subtypes with higher uptake, such as thick-walled or mural nodule types, differentiation from inflammatory, infectious, or granulomatous diseases, which can also exhibit high FDG uptake, remains difficult (60, 61). Diagnostic thresholds and the prognostic value of FDG uptake in LCCA have yet to be clearly established, limiting its role in precision assessment. Nonetheless, PET/CT still holds promise in evaluating tumor metabolism and biological behavior, drawing reference from management strategies used for low-metabolism pure ground-glass nodules (57, 62). Of note, a study by Jung et al. (39) observed fibrotic changes along the cyst wall margins, with increasing fibrosis seen in more advanced imaging phases. This finding suggests that novel tracers such as FAPI (fibroblast activation protein inhibitor) may overcome the limitations of traditional FDG imaging and offer more accurate early diagnosis for LCCA, an area worthy of further exploration. At the same time, innovations in imaging and multimodal integration offer new opportunities for the diagnosis and management of LCCA. MRI, with its excellent soft tissue resolution and multifunctional imaging capabilities, provides unique advantages in evaluating lung diseases. Techniques such as diffusion-weighted imaging (DWI) and DCE-MRI may theoretically help characterize tumour biology and guide personalized treatment (63, 64). Radiomics enables high-throughput extraction of morphological and textural features from CT images. These features can be integrated into a radiomic phenotype that reflects tumor biology, allowing more accurate prediction of diagnosis, prognosis, and treatment response in LCCA (65–67). Furthermore, the complex tumor microenvironment of LCCA, which may involve inflammation, necrosis, and fibrosis, presents an opportunity for habitat imaging analysis. By identifying spatially heterogeneous habitats associated with specific radiomic phenotypes, it is expected to reveal molecular mechanisms underlying LCCA invasiveness and progression, ultimately helping to refine prognostic prediction frameworks.

To enhance clinical applicability, a stepwise diagnostic pathway is proposed: HRCT as the first−line tool; serial CT follow−up for indeterminate lesions; and, when needed, PET/CT as a supplementary modality. Emerging techniques such as radiomics, habitat imaging, and novel tracers remain investigational but may further improve lesion characterization and prediction of invasiveness in the future. This prioritization ensures that clinically feasible approaches are applied before more advanced or experimental methods.

In the era of precision lung cancer screening as articulated by Chen et al., LCCA management should move beyond one-size-fits-all approaches. Drawing on the established follow−up strategies for subsolid nodules, we propose a risk-stratified pathway based on (1): baseline CT morphology (Shen type, wall thickness, nodule size); (2) dynamic change (growth pattern of cystic vs. solid components); and (3) patient factors (age, smoking history, emphysema burden). For low-risk lesions (Shen I, wall thickness <2 mm, no mural nodule), 12-month LDCT follow-up is appropriate. For intermediate-risk lesions (Shen II, wall thickness 2–4 mm, or small mural nodule <5 mm), 6-month follow-up. For high-risk lesions (Shen III/IV, or any lesion with ≥8 mm solid component, or lymph node suspicion), immediate diagnostic workup (PET/CT, biopsy, or clinical consultation) is recommended (68, 69).

**Figure 5:**
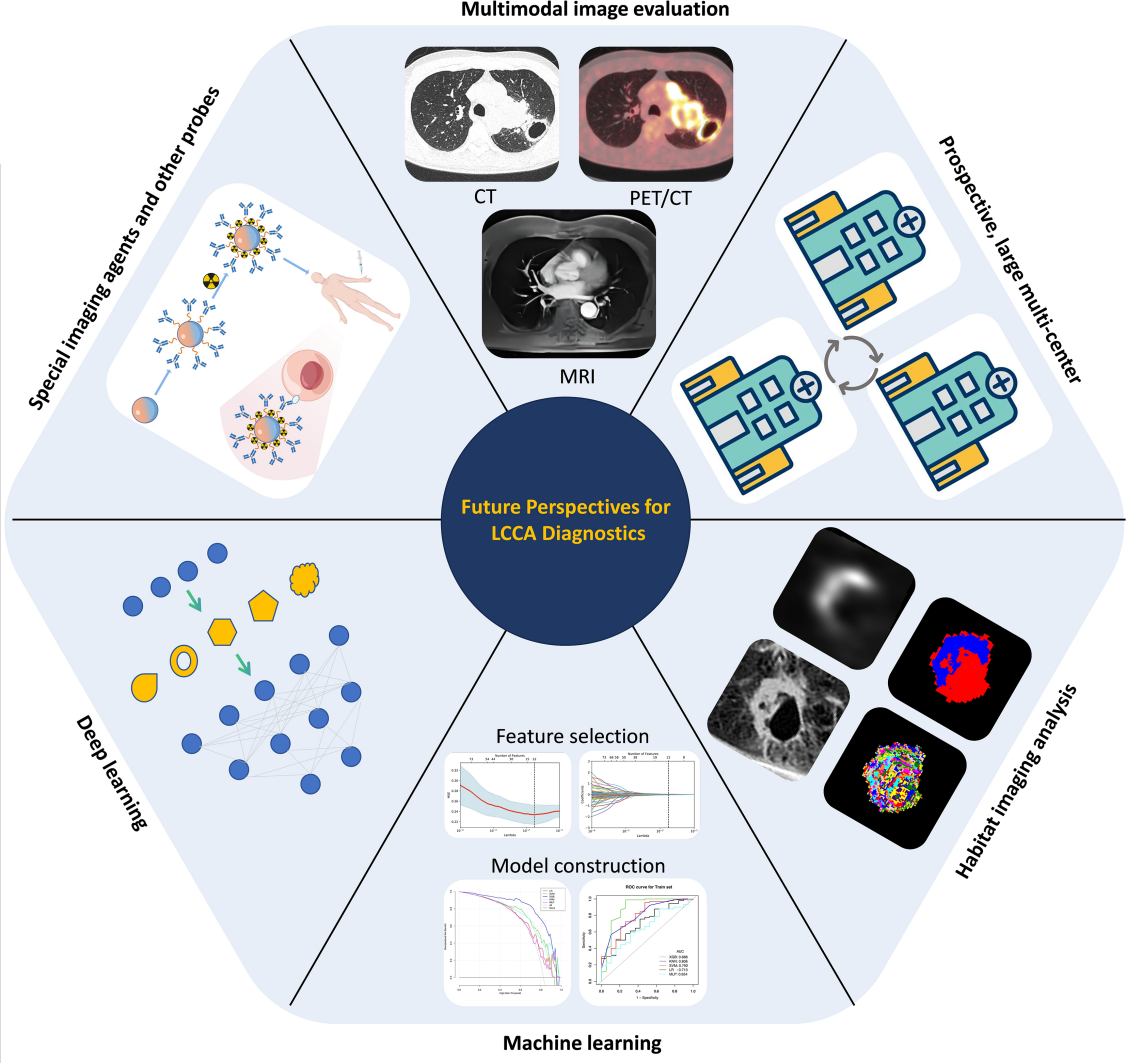
Future perspectives for LCCA diagnostics. Special imaging agents and other probes was created with (https://BioGDP.com).

Finally, the development of standardized management guidelines for LCCA relies on the accumulation of high-quality clinical data. However, most current studies are single-center and retrospective in nature, lacking support from multimodal imaging integration and large-scale prospective datasets. These limitations hinder progress toward establishing standardized protocols. In addition, the 2017 Fleischner Society guidelines lack targeted recommendations for LCCA, making it necessary to upgrade and revise the current guidelines to include LCCA-specific evaluation and management standards (70). Future efforts should focus on prospective cohort studies or large multi-center investigations to develop stratified management pathways based on integrated imaging, pathological, and molecular characteristics. Special attention should be given to refining the classification of LCCA within the Lung-RADS system and improving risk assessment models, ultimately leading to the establishment of standardized clinical guidelines for LCCA management. Future perspectives for LCCA diagnostics are shown in **Figure 5**.

In conclusion, imaging research on LCCA must evolve from single-dimensional morphological analysis to a comprehensive integration of morphology, function, and molecular characteristics. Addressing key issues, such as the lack of standardized classification systems, limitations of conventional imaging techniques, and the absence of prospective data, is essential for establishing a comprehensive management framework encompassing early diagnosis, invasiveness prediction, treatment decision-making, and prognostic assessment. Achieving this goal will require not only the application of advanced tools such as radiomics, habitat imaging, and novel molecular tracers, but also interdisciplinary collaboration and support from large-scale data resources. These efforts will enable the development of precise, personalized diagnostic and therapeutic strategies for LCCA patients and provide a foundation for improving lung cancer clinical guidelines and optimizing clinical decision-making.
